# Supplementary material for: Circulating microRNA Biomarker for Detecting Breast Cancer in High-Risk Benign Breast Tumors
Source: Int J Mol Sci. 2023 Apr 20;24(8):7553. doi: 10.3390/ijms24087553 (PMC10142546; doi:10.3390/ijms24087553)

The Area Under the Curve (AUC) Plots

1. miRBase ID: hsa-mir-215-5p

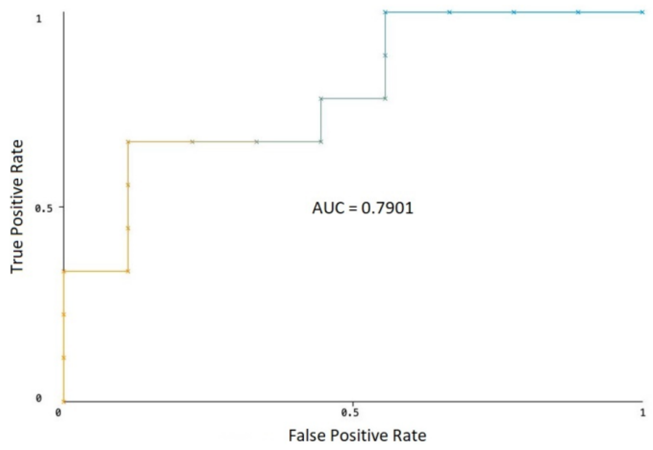

2. miRBase ID: hsa-mir-200a-3p

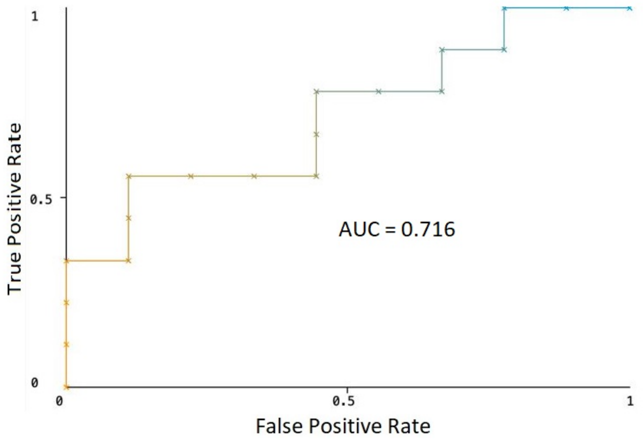

3. miRBase ID: hsa-mir-141-3p

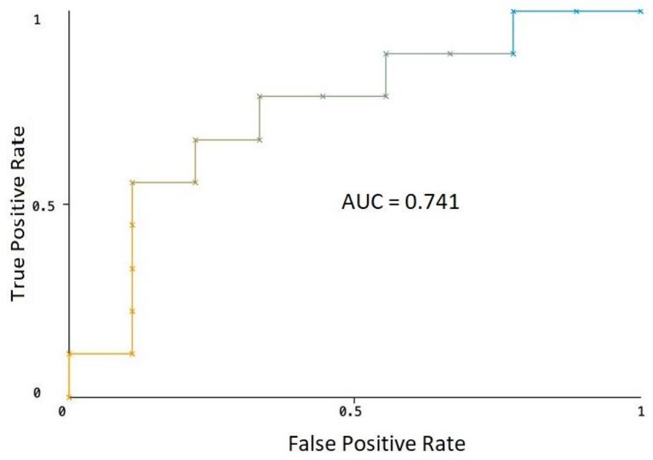

4. miRBase ID: hsa-mir-215-5p and hsa-mir-200a-3p

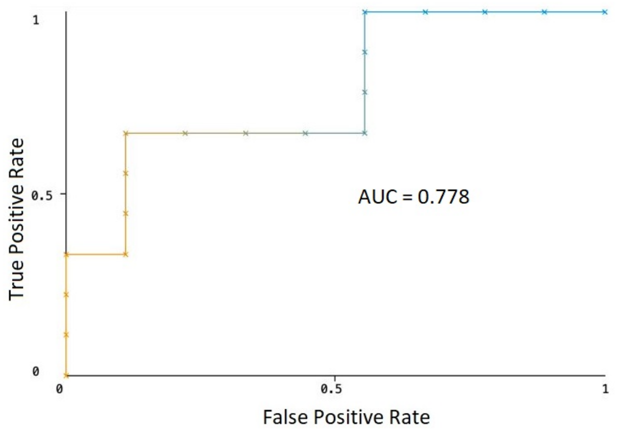

5. miRBase ID: hsa-mir-215-5p and hsa-mir-141-3p

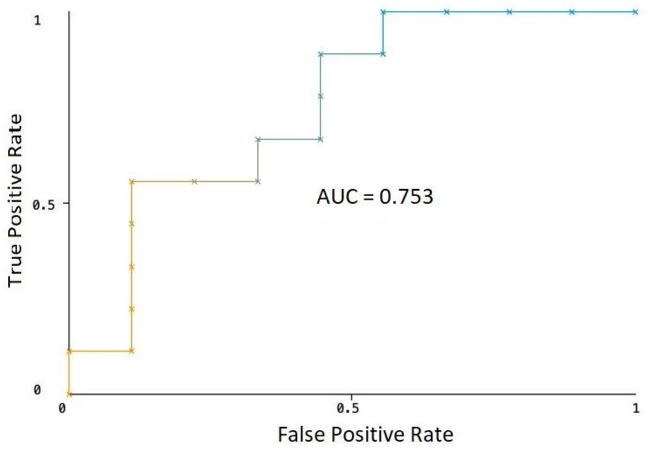

6. miRBase ID: hsa-mir-128-3p

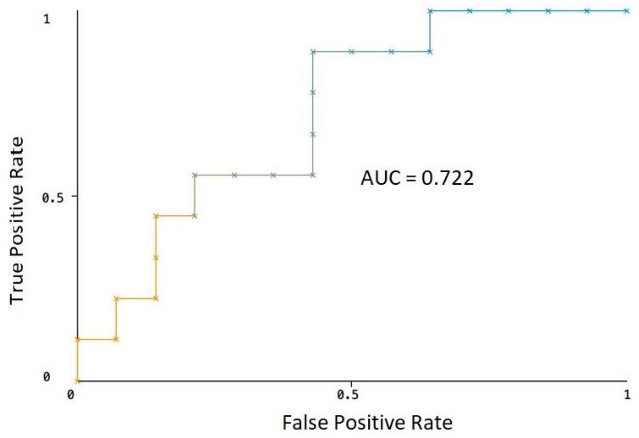

7. miRBase ID: hsa-mir-130b-5p

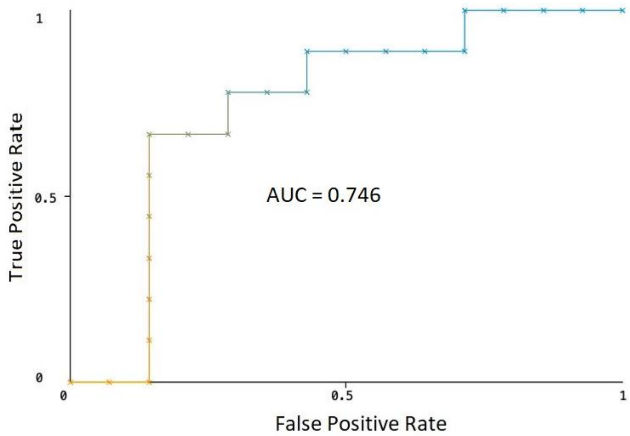

8. miRBase ID: hsa-mir-28-5p

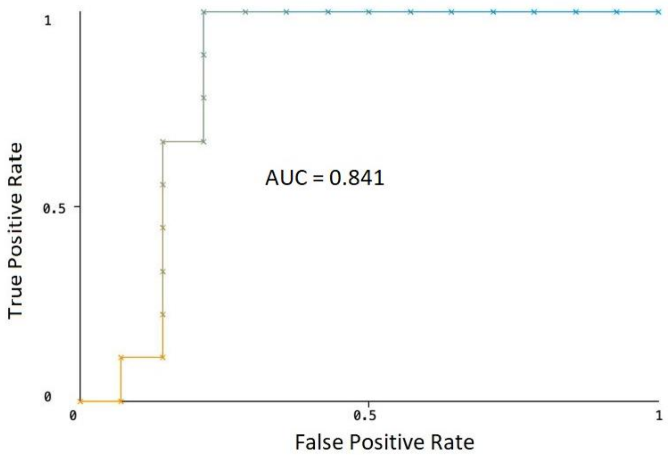

9. miRBase ID: hsa-mir-421

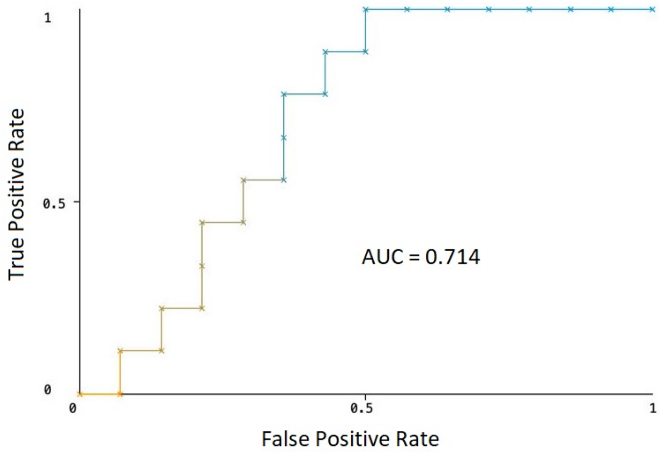

10. miRBase ID: hsa-mir-28-5p and hsa-mir-421

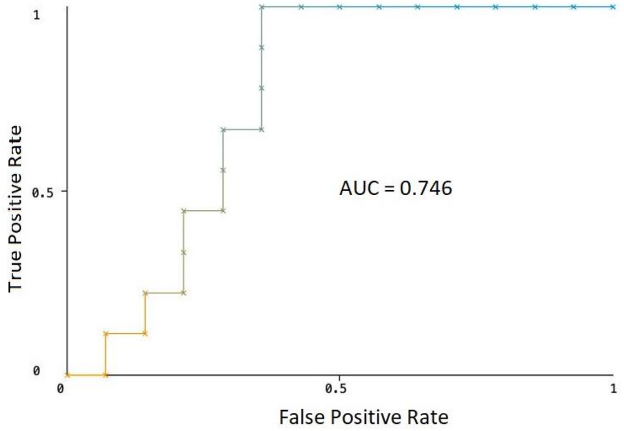

11. miRBase ID: hsa-mir-130b-5p, hsa-mir-28-5p and hsa-mir-421

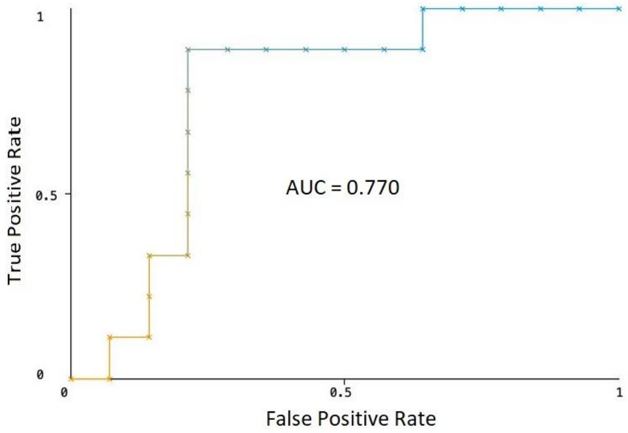

Supplement: Supplementary file 1 [file ijms-24-07553-s001.zip › Supplementary Figure S1. The Area Under the Curve.pdf]
