# Supplementary material for: Circulating microRNA Biomarker for Detecting Breast Cancer in High-Risk Benign Breast Tumors
Source: Int J Mol Sci. 2023 Apr 20;24(8):7553. doi: 10.3390/ijms24087553 (PMC10142546; doi:10.3390/ijms24087553)
Supplement: Supplementary file 1 [file ijms-24-07553-s001.zip › Supplementary Table S1. Sample information_mj.pdf]

Supplementary Table S1. Sample information

Case ID	Gender	Race	BMI	Smoking Status	Age at Sample Acquisition	Histologic Type	Histologic Category	
HB1	F	White	34	Never	51	Atypical Ductal Hyperplasia	ADH	High-risk benign
HB2	F	White	23	Never	67	Atypical Ductal Hyperplasia	ADH	
HB3	F	White	24	Former	70	Atypical Ductal Hyperplasia involving papilloma	ADH	
HB4	F	White	29	Former	61	Atypical Lobular Hyperplasia	ALH	
HB5	F	White	33	Never	65	Atypical Lobular Hyperplasia and papilloma	ALH	
HB6	F	White	25	Never	51	Flat Epitherial Atypia, fibrocystic changes, usual ductal hyperplasia	FEA	
HB7	F	White	21	Never	45	Flat Epitherial Atypia, focal microcalcifications, fibrocystic change, focal usual ductal hyperplasia	FEA	
HB8	F	White	31	Former	61	Focal Atypical Lobular Hyperplasia	ALH	
HB9	F	White	22	Never	73	Focal Atypical Lobular Hyperplasia and focal ductal hyperplasia with microcalcifications	ALH	
HB10	F	White	23	Never	50	Focal Flat Epitherial Atypia	FEA	
HB11	F	White	19	Never	51	Focal Flat Epitherial Atypia, focal radial scar, usual ductal hyperplasia	FEA	
HB12	F	White	30	Never	49	Atypical Ductal Hyperplasia	ADH	
HB13	F	White	26	Never	48	Multifocal Flat Epitherial Atypia	FEA	
HB14	F	White	NA	NA	47	Right breast Atypical Ductal Hyperplasia, Left breast Atypical Lobular Hyperplasia	ADH	
MB1	F	White	35	Never	65	Benign breast parenchyma, columnar cell change, usual ductal hyperplasia, intraductal papilloma	Intraductal papilloma	Moderate-risk benign
MB2	F	White	24	Never	62	Fibrocystic changes, usual ductal hyperplasia, small intraductal papilloma	Intraductal papilloma	
MB3	F	White	20	Never	38	Radial scar, sclerosing adenosis, fibrocystic changes, focal duct hyperplasia	Radial scar	
MB4	F	White	32	Current	72	Small intraductal papilloma	Intraductal papilloma	
Be1	F	White	20	Never	52	Benign breast tissue, stromal fibrosis, small fibroadenomas	FA	No-risk benign
Be2	F	White	30	Former	63	Benign breast tissue, focal fibrocystic changes	Fibrocystic change	
Be3	F	White	29	Former	42	Fibroadenoma	FA	
Be4	F	White	NA	Former	50	Fibroadenoma	FA	
Be5	F	White	NA	Former	54	Fibroadenoma	FA	
Be6	F	White	NA	NA	63	Fibroadenoma	FA	
Be7	F	White	23	Never	41	Fibrocystic changes, fibroadenomatiod mastopathy, focal microcalcifications	Fibrocystic change	
Be8	F	White	31	Never	65	fibrocystic change, microcalcifications	Fibrocystic change	
Be9	F	White	23	Former	58	Microcalcifications, fibrocystic changes, benign ducts and lobules	Fibrocystic change	

Case ID	Gender	Race	BMI	Smoking Status	Age at Sample Acquisition	Histologic Type	Histologic Category	pTNM-Stage	pT	pN	pM	Grade	ER	PR	HER2	Subtype
CA1	F	White	30	Current	42	Invasive ductal carcinoma, NOS	IDC	I	pT1a	pN0	pM: NA	Grade 1	Pos	Pos	Neg	LA
CA2	F	White	29	Former	62	Invasive ductal carcinoma, NOS	IDC	I	pT1a	pN0	pM: NA	Grade 1	Pos	Pos	Neg	LA
CA3	F	White	38	Former	47	Invasive papillary carcinoma	IDC	I	pT1a	pN0(sn)	pM: NA	Grade 1	Pos	Pos	Neg (1+)	LA
CA4	F	White	32	Never	67	Invasive ductal carcinoma with metaplastic features	MC	I	pT1b	pN0	pM: NA	Grade 3	Neg	Neg	Neg	TN
CA5	F	White	33	Current	40	Metaplastic carcinoma, spindle cell type	MC	I	pT1a	pN0	pM: NA	Grade 3	Neg	Neg	Neg	TN
CA6	F	White	30	Former	66	Invasive ductal carcinoma, NOS	IDC	I	pT1b	pN0(sn)	pM: NA	Grade 1	Pos	Pos	Neg	LA
CA7	F	White	29	Former	64	Invasive ductal carcinoma, NOS	IDC	I	pT1b	pN0(sn)	pM: NA	Grade 3	Neg	Neg	Neg	TN
CA8	F	White	18	Former	55	Invasive lobular carcinoma	ILC	I	pT1b	pN0	pM: NA	Grade 1	Pos	Pos	Neg	LA
CA9	F	White	21	Former	61	Ductal carcinoma *in situ*	DCIS	0	ypTis	ypN0	pM: NA	NA	Neg	Neg	Neg	TN

Note: CA9 was treated with neoadjuvant therapy after blood collection.

(shaded box): Sample used for proteomics.
