# Supplementary material for: Circulating microRNA Biomarker for Detecting Breast Cancer in High-Risk Benign Breast Tumors
Source: Int J Mol Sci. 2023 Apr 20;24(8):7553. doi: 10.3390/ijms24087553 (PMC10142546; doi:10.3390/ijms24087553)
Supplement: Supplementary file 1 [file ijms-24-07553-s001.zip › Supplementary Table S3. Association between miRNA and its target genes_mj.pdf]

Supplementary Table S3. Association between miRNA and its target genes

MirGeneDB ID	CA		HB	
	Positive	Negative	Positive	Negative
Hsa-Mir-10-P2c_5p	*PTPRJ*		*F13A1, HABP2*	*TUBB*
Hsa-Mir-122_5p	*C1RL, PTPRJ, ANTXR1*	*YWHAQ*	*F13A1, TUBA4A*	
Hsa-Mir-128-P1_3p		*KRT2, ITGB3, LAMB1, RAP1B, CTSS*	*LTF*	*YWHAQ*
Hsa-Mir-130-P1c_3p	*MYH9, IGFBP3, CNTN1*	*ALDOA*	*LAMB1*	
Hsa-Mir-130-P4a_5p		*PZP*		*LTF*
Hsa-Mir-15-P1a_5p	*SPTB*	*KRT10, KRT2, CTSS*		*FLNA*
Hsa-Mir-15-P1c_5p	*HBG2*	*ATRN, HABP2, MEGF8*		*TGFBI, ZYX*
Hsa-Mir-17-P4a_5p		*C1RL, LAMB1, NRP1*		*HABP2*
Hsa-Mir-185_5p		*PGLYRP2, C1RL, LAMB1, SPARCL1*	*SPTB, LTF*	*ITGB3, ACTBL2, IGF2R*
Hsa-Mir-19-P2a_3p	*PZP*	*KRT2, LAMB1*		*FLNA, TGFBI*
Hsa-Mir-19-P2c_3p	*PZP*	*KRT2, LAMB1*		*FLNA, TGFBI*
Hsa-Mir-28-P1_5p		*PZP*	*VCL, HSPA8, ITGB3, PKM, ACTBL2, IGF2R*	*SPTB, LTF, NRP1*
Hsa-Mir-877_3p*	*LAMB1, NRP1*			*LTF, SELENBP1*
Hsa-Mir-885_5p	*LCP1, C1RL, PLXDC2, ANTXR1*	*PKM*	*TUBA4A, IGF1*	*HSPA8, PKM*
Hsa-Mir-95-P2_3p	*CNTN1*	*NRP1*	*SPTB, ITGB3, LTF*	*FLNA, PKM, ACTBL2*
