# Supplementary material for: Circulating microRNA Biomarker for Detecting Breast Cancer in High-Risk Benign Breast Tumors
Source: Int J Mol Sci. 2023 Apr 20;24(8):7553. doi: 10.3390/ijms24087553 (PMC10142546; doi:10.3390/ijms24087553)
Supplement: Supplementary file 1 [file ijms-24-07553-s001.zip › Supplementary Table S4r. Information of the Identified miRNAs.pdf]

Supplementary Table S4. Information of the Identified miRNAs

MirGeneDB	miRBase	Family	Chromosome	Seed	Sequences of identified biomarkers	Note
Hsa-Mir-10-P2c_5p	hsa-mir-99a-5p	MIR-10	21	ACCCGUA		
Hsa-Mir-122_5p	hsa-mir-122-5p	MIR-122	18	GGAGUGU		
**Hsa-Mir-128-P1_3p**	**hsa-mir-128-3p**	**MIR-128**	**3**	**CACAGUG**	ucacagugaaccggucucuuu	
Hsa-Mir-130-P1c_3p	hsa-mir-130a-3p	MIR-130	11	AGUGCAA		
**Hsa-Mir-130-P4a_5p**	**hsa-mir-130b-5p**	**MIR-130**	**22**	**CUCUUUC**	acucuuucccuguugcacuac	
Hsa-Mir-15-P1a_5p	hsa-mir-15a-5p	MIR-15	13	AGCAGCA		
Hsa-Mir-15-P1c_5p	hsa-mir-424-5p	MIR-15	X	AGCAGCA		
Hsa-Mir-17-P2a_5p	hsa-mir-18a-5p	MIR-17	13	AAGGUGC		
Hsa-Mir-17-P4a_5p	hsa-mir-20a-5p	MIR-17	13	AAAGUGC		
Hsa-Mir-185_5p	hsa-mir-185-5p	MIR-185	22	GGAGAGA		
**Hsa-Mir-192-P1_5p**	**hsa-mir-215-5p**	**MIR-192**	**1**	**UGACCUA**	augaccuaugaauugacagac	
Hsa-Mir-19-P2a_3p	hsa-mir-19b-3p (19b-1)	MIR-19	13	GUGCAAA		
Hsa-Mir-19-P2c_3p	hsa-mir-19b-3p (19b-2)	MIR-19	X	GUGCAAA		
**Hsa-Mir-28-P1_5p**	**hsa-mir-28-5p**	**MIR-28**	**3**	**AGGAGCU**	aaggagcucacagucuauugag	
Hsa-Mir-3613_5p	hsa-mir-3613-5p	MIR-3613	13	GUUGUAC		
Hsa-Mir-361-v1_3p*	hsa-mir-361-3p	MIR-361	X	UAUCAGA		
Hsa-Mir-362-P1_5p	hsa-mir-362-5p	MIR-362	X	AUCCUUG		
Hsa-Mir-877_3p*	hsa-mir-877-3p	MIR-877	6	UAGAGGA		
Hsa-Mir-885_5p	hsa-mir-885-5p	MIR-885	3	CCAUUAC		
**Hsa-Mir-8-P2a_3p**	**hsa-mir-200a-3p**	**MIR-8**	**1**	**AACACUG**	uaacacugucugguaa cgaugu	miR-200 family
**Hsa-Mir-8-P2b_3p**	**hsa-mir-141-3p**	**MIR-8**	**12**	**AACACUG**	uaacacugucugguaa agaugg	miR-200 family
**Hsa-Mir-95-P2_3p**	**hsa-mir-421**	**MIR-95**	**X**	**UCAACAG**	aucaacagacauuaauugggcgc	
